# Can the sustainable development goals reduce the burden of nutrition-related non-communicable diseases without truly addressing major food system reforms?

**DOI:** 10.1186/s12916-015-0383-7

**Published:** 2015-06-16

**Authors:** Corinna Hawkes, Barry M. Popkin

**Affiliations:** Centre for Food Policy, School of Arts & Social Sciences, City University London, Northampton Square, London, EC1V 0HB UK; Department of Nutrition SPH and Carolina Population Center, University of North Carolina, CB#8120, 137 E. Franklin St., Chapel Hill, NC 27516 USA

**Keywords:** Sustainable development goals, Diet, Nutrition transition, Food system, Processed food, non-communicable diseases

## Abstract

While the Millennium Development Goals (MDGs; 2000–2015) focused primarily on poverty reduction, hunger and infectious diseases, the proposed Sustainable Development Goals (SDGs) and targets pay more attention to nutrition and non-communicable diseases (NCDs). One of the 169 proposed targets of the SDGs is to reduce premature deaths from NCDs by one third; another is to end malnutrition in all its forms. Nutrition-related NCDs (NR-NCDs) stand at the intersection between malnutrition and NCDs. Driven in large part by remarkable transformations of food systems, they are rapidly increasing in most low and middle income countries (LMICs). The transformation to modern food systems began in the period following World War II with policies designed to meet a very different set of nutritional and food needs, and continued with globalization in the 1990s onwards. Another type of food systems transformation will be needed to shift towards a healthier and more sustainable diet – as will meeting many of the other SDGs. The process will be complex but is necessary. Communities concerned with NCDs and with malnutrition need to work more closely together to demand food systems change.

## Background

After years of discussion and debate, the Sustainable Development Goals (SDGs) and targets will be adopted at the UN General Assembly in September (Box 1) [1]. The SDGs replace the Millennium Development Goals (2000–2015) (MDGs, Box 2) as the world’s framework for development [2]. The designers of the SDGs clearly had something different in mind to the MDGs. There are a lot more of them – 17 SDGs (Box 1) relative to 8 MDGs (Box 2), and 169 targets relative to 18; they are global in scope – unlike the focus of the MDGs on the developing world only; and they are concerned with global public goods, not just nationally defined problems. Given the far greater number, it is not surprising there are also important content differences. Non-communicable diseases (NCDs) and nutrition constitute two such differences. The MDGs ignored NCDs: they were a non-disease, with all the emphasis placed on infectious diseases. In contrast, Goal 3 of the SDGs (Box 1) includes the target *to reduce by one third, by 2030, premature mortality from NCDs through prevention and treatment and promote mental health and well-being* [1].

Nutrition was also given short shrift in the MDGs with reference only to underweight. In the SDGs, there is a now a target (2.2) to end malnutrition in all its forms under Goal 2 (Box 1). This commentary focuses on the intersection between these health concerns – nutrition-related NCDs (NR-NCDs) – and what needs to be done to move towards achieving both the NCD and nutrition targets.

### The nutrition transition and the transformed global food system

The world has been moving headlong towards an unhealthy and equally unsustainable pattern of food production and consumption for decades. Often termed the “nutrition transition”, these changes have taken hold more recently – but more rapidly – in low and middle income countries (LMICs). As widely reported on elsewhere, e.g. [3], the nutrition transition is characterized by a series of typical changes. People consume more packaged, processed foods; the content of foods is increasingly laden with added sugar, particularly in beverages, as well as salt and refined carbohydrate; foods are more often prepared – both in the home and out – with larger quantities of vegetable oils; the number of eating events has grown from the traditional two to three meals per day to numerous snacking events; in those populations with income growth, food intake from animal sources has grown rapidly; consumption of fruits and vegetables, legumes and many healthful coarse grains and root crops has declined or remains woefully inadequate. While these characteristics are clear, they are not the same everywhere. The reality is a complex picture of a heterogeneous mix of trends between foods and countries [4].

Diets are not all that have changed. Concurrently there have been remarkable declines in physical activity at work and home and in transport and leisure, as modern technology has rapidly shifted into all sectors of life globally [5]. As a result it is estimated that over 12 million deaths in 2010 were attributable to poor diets and physical inactivity [6].

How to change? One important focus of action needs to be the global food system ([7], Anand et al. Global food consumption and its impact on cardiovascular disease requires global solutions with a focus on the globalized food system (unpublished)). The modern, global food system emerged from policies and practices designed to meet a very different set of nutritional and food needs. In the post World War II period, government policies focused on increasing production of a small number of key commodities (e.g., corn and soybeans followed by animal foods) as a means of boosting intake of calories and protein. Moving on, policies encouraged the “globalization” of the food system [8]. What followed was greater participation and control in the food system by private sector players – such as food manufacturers and supermarkets – who purchase food directly from farmers and transform it in various ways for consumption [9]. These policies were designed to, among other goals, address under-nutrition and national and global food insecurity, without the knowledge – or foresight – of what they held in store for future health needs.

Although these changes are uneven within regions and even within countries, all LMICs have been affected by these shifts. We now face a highly distorted system which fails to put quality protein and diversity into the diets of the poor while “succeeding” in feeding people large quantities of refined carbohydrates and highly processed foods [10]. In turn, the world faces a huge public health burden from NR-NCDs. It is increasingly evident that consumption of highly processed foods based mainly on refined carbohydrates, excessive sodium, added sugar and saturated fat, is linked with increased risk of obesity and diabetes [11].

### Future directions and conclusions

No country is untouched by the negative health impacts of poor quality diets [12]. Countries are beginning to take actions to promote healthier eating at the national level [13] but few are truly taking on the broader food system, its priorities, and its entire structure. Yet meeting the nutrition and NCD targets in the SDGs – as well as many other targets -- will take a concerted level of focus and political will to do so.

It is therefore regrettable that the SDG’s hardly focus on the long term challenge of creating healthier, more sustainable food systems. While Goal 12 on sustainability (Box 1) includes some relevant targets, transforming the food system will take multiple actions at multiple levels, from local level innovations to enhancing food access for vulnerable groups to the restructuring of global level governance of agriculture, food, nutrition and health.

One action that academics and civil society can take to further a political environment more amenable to these changes is to engage with colleagues concerned with different forms of malnutrition, including NR-NCDs. We need greater unity across malnutrition in all its forms. We know that factors such as high quality diets for pregnant women, breastfeeding, appropriate complementary foods, healthy, nutritious foods in infancy and beyond, access to good healthcare and hygienic environments, are needed to address both under-nutrition and NR-NCDs [12]. We know that the global food system shifts are affecting both sets of problems. The nutrition and NCD communities should therefore come together to provide evidence and advocate for healthy food policies and healthier food systems. This demand for change – along with more and better evidence for the changes needed and how they can happen – is necessary if the world is going to come anywhere close to attaining the aspirations set by the SDGs.

## Box 1. The proposed SDGs

Goal 1: End poverty in all its forms everywhere

Goal 2: End hunger, achieve food security and improved nutrition and promote sustainable agriculture

Goal 3: Ensure healthy lives and promote well-being for all at all ages

Goal 4: Ensure inclusive and equitable quality education and promote lifelong learning opportunities for all

Goal 5: Achieve gender equality and empower all women and girls

Goal 6: Ensure availability and sustainable management of water and sanitation for all

Goal 7: Ensure access to affordable, reliable, sustainable and modern energy for all

Gal 8: Promote sustained, inclusive and sustainable economic growth, full and productive employment and decent work for all

Goal 9: Build resilient infrastructure, promote inclusive and sustainable industrialization and foster innovation

Goal 10: Reduce inequality within and among countries

Goal 11: Make cities and human settlements inclusive, safe, resilient and sustainable

Goal 12: Ensure sustainable consumption and production patterns

Goal 13: Take urgent action to combat climate change and its impacts*

Goal 14: Conserve and sustainably use the oceans, seas and marine resources for sustainable development

Goal 15: Protect, restore and promote sustainable use of terrestrial ecosystems, sustainably manage forests, combat desertification, and halt and reverse land degradation and halt biodiversity loss

Goal 16: Promote peaceful and inclusive societies for sustainable development, provide access to justice for all and build effective, accountable and inclusive institutions at all levels

Goal 17: Strengthen the means of implementation and revitalize the global partnership for sustainable development

Source: [1]

* taking into account ongoing World Trade Organization (WTO) negotiations and WTO Doha Development Agenda and Hong Kong Ministerial Mandate

## Box 2. The Millennium Development Goals (2000–2015)

1. To eradicate extreme poverty and hunger
2. To achieve universal primary education
3. To promote gender equality
4. To reduce child mortality
5. To improve maternal health
6. To combat HIV/AIDS, malaria, and other diseases
7. To ensure environmental sustainability
8. To develop a global partnership for development

Source: [2]
